# Association of sleep and glycaemic status with all-cause mortality: a prospective cohort study

**DOI:** 10.7189/jogh.16.04002

**Published:** 2026-01-12

**Authors:** Andi Xu, Cao Zhi, Sicong Wang, Fei Cai, Zuhui Zhang, David Ta-Wei Chu, Wenyuan Li, Chi Pang Wen, Xifeng Wu

**Affiliations:** 1 Center of Clinical Big Data and Analytics of The Second Affiliated Hospital, and School of Public Health, Zhejiang University School of Medicine, Hangzhou, China; 2 The Key Laboratory of Intelligent Preventive Medicine of Zhejiang Province, Hangzhou, China; 3 MJ Health Management Center, Taipei, Taiwan; 4 National Institute for Data Science in Health and Medicine, Zhejiang University, Hangzhou, Zhejiang, China; 5 School of Medicine and Health Science, George Washington University, Washington DC, USA

## Abstract

**Background:**

Suboptimal sleep and diabetes are major contributors to mortality. However, whether sleep patterns differentially affect mortality across glycaemic statuses remains unclear. This study examined associations of sleep patterns (sleep duration and sleep disorders) with all-cause mortality among individuals with normoglycaemia, prediabetes, and diabetes.

**Methods:**

Data were obtained from the Taiwan MJ cohort, including 534 238 participants enrolled between 1996 and 2022. Sleep duration (‘less than 6 hours’, ‘6–8 hours’, ‘more than 8 hours’) and sleep disorders (yes/no) were assessed via standardised questionnaires. Glycaemic status was classified as normoglycaemia, prediabetes, or diabetes. Mortality data were obtained from the Taiwan Death Registry. Cox proportional hazards regression models were employed to evaluate the association between sleep patterns and the risk of all-cause mortality.

**Results:**

The study included 363 863 participants with normoglycaemia, 144 602 with prediabetes, and 25 773 with diabetes. Over a median follow-up period of 19 years, 52 208 deaths were recorded. Compared with those who slept 6–8 hours, normoglycaemia individuals who slept less than 6 hours had a higher risk of all-cause mortality (hazard ratio (HR) = 1.05; 95% confidence interval (CI) = 1.02–1.08) and those who slept more than eight hours had a higher risk of all-cause mortality across all glycaemic groups: normoglycaemia (HR = 1.19; 95% CI = 1.15–1.24), prediabetes (HR = 1.24; 95% CI = 1.19–1.30), and diabetes (HR = 1.29; 95% CI = 1.22–1.36). Sleep disorders were also associated with increased mortality among individuals with prediabetes (HR = 1.04; 95% CI = 1.01–1.07) and diabetes (HR = 1.07; 95% CI = 1.02–1.11).

**Conclusions:**

Long sleep durations and sleep disorders were associated with increased mortality, especially among individuals with impaired glucose regulation while short sleep duration was discovered to associate with increased risk of mortality in people with normoglycaemia. These findings highlight the potential role of sleep assessment in risk stratification, although the observational nature of the study limits causal inference.

Suboptimal sleep is a growing global health concern. A substantial proportion of adults worldwide fail to meet the recommended sleep duration guidelines. Studies have shown that many individuals do not get an adequate amount of sleep [1,2]. For instance, approximately 24.5% [1] of people in the Netherlands, the USA, and the UK sleep fewer hours than recommended. Sleep disorders [3], encompassing a broad spectrum of conditions ranging from insomnia to circadian rhythm sleep-wake disorders, also affect a significant portion of the population [4,5]. Previous evidence has demonstrated that inappropriate sleep duration, sleep disturbances, and sleep disorders are associated with increased risks of mortality [6–10].

Meanwhile, the prevalence of diabetes and prediabetes has been rising, placing a considerable burden on individuals, families, and society. According to the WHO, in 2022, 830 million people lived with diabetes, which has become one of the leading causes of death [11], shortening life expectancy by several years [12,13]. In the USA, for example, an estimated 38.0% of adults had prediabetes in 2021 [14]. Meanwhile, prediabetes, a major risk factor for diabetes and a type of glucose impairment, is also becoming increasingly common worldwide, and this condition has been independently linked to higher risks of mortality [15].

Previous studies have investigated the relationship between sleep and glycaemic status (prediabetes and diabetes) mostly in the Western populations. It is well established that individuals with diabetes exhibit altered sleep patterns [16,17] compared to the general population. Poor sleep behaviour adversely affects the health [18] of diabetic patients and is associated with increased mortality risk [19,20] among this group. However, whether the association between sleep and all-cause mortality differs across the spectrum of glycaemic status, including normoglycaemia, prediabetes, and diabetes, remains uncertain. In particular, research focusing on individuals with prediabetes is limited, despite this group being at elevated metabolic risk. Furthermore, most existing studies have been conducted in Western populations, with relatively few large-scale investigations in Asian cohorts. Therefore, this study aims to investigate the associations between sleep patterns, including sleep duration and sleep disorders, and the risk of all-cause mortality among individuals with normoglycaemia, prediabetes and diabetes in a large prospective cohort of Taiwanese adults.

## METHODS

### Study population

This study was based on the MJ cohort, which has recruited over half a million individuals in Taiwan since 1996. Each participant completed a self-administered questionnaire that included a range of questions related to demographics, lifestyle, medical conditions, and family history, and underwent standardised general physical examinations, anthropometric measurements, and biochemical tests. Detailed descriptions of the MJ cohort have been published elsewhere [21]. All participants provided written informed consent.

**Figure 1:**
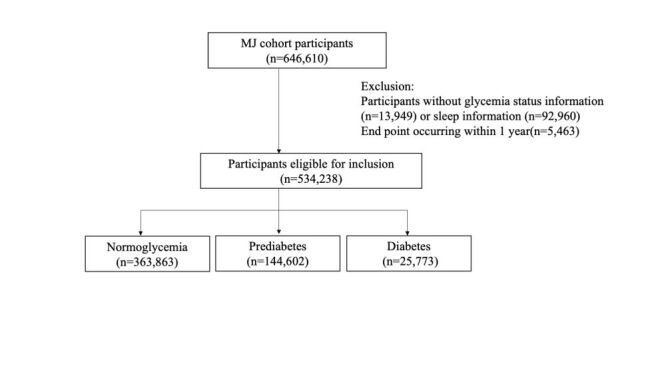
Flowchart of participant selection.

A total of 646 610 individuals were enrolled in this study. After excluding those without glycaemic status data (n = 13 949), without information on sleep behaviour (n = 92 960), and those whose endpoints occurred within one year of enrolment (n = 5463), the final analytic sample consisted of 534 238 participants. The detailed process of inclusion and exclusion is illustrated in **Figure 1**. Participants were followed until 31 December 2022, or until death, whichever occurred first.

### Ascertainment of sleep

Sleep patterns were assessed based on sleep duration and the presence of sleep disorders. Participants reported their sleep information using standardised self-administered questionnaires. For sleep duration, each participant was asked, ‘How many hours do you usually sleep a day?’ [22]. Daily sleep duration was categorised as ‘less than 6 hours’, ‘6–8 hours’, and ‘more than 8 hours.’ Regarding sleep disorders, participants were asked: ‘How did you evaluate your sleep quality last month?’. Response options included: use of sleeping pills or drugs, difficulty falling asleep, can fall asleep but easily awake, dreamy sleep, and sleep well. Those who answered ‘sleep well’ were classified as not having a sleep disorder, while any of the other responses were considered indicative of a sleep disorder.

### Ascertainment of glycaemic status

Glycaemic status was defined according to the criteria established by the American Diabetes Association [23]. In the morning, an overnight fasting blood samples were taken and plasma glucose concentrations were measured using an automatic biochemical analyser (7150; Hitachi) in accordance with the laboratory’s standardised procedures. Participants were categorised as having normoglycaemia, prediabetes, or diabetes. Individuals with fasting blood glucose levels below 100 mg/dL, without a diagnosis of diabetes or use of antidiabetic medication, were classified as normoglycemic. Those with fasting blood glucose levels between 100 and 125 mg/dL, in the absence of a diabetes diagnosis or antidiabetic medication use, were defined as having prediabetes. Participants with fasting blood glucose levels above 125 mg/dL, or who reported having diabetes or using antidiabetic medication, were classified as having diabetes.

### Ascertainment of outcome

The primary outcome of this study was all-cause mortality. Mortality data, including exact dates of death, were obtained from the Taiwan Death Registry. Participants were followed until death or 31 December 2022, whichever came first.

### Covariates

This study incorporated a range of potential covariates, including sociodemographic characteristics, clinical variables, and lifestyle factors. Demographic information included sex, age, marital status (married, single, divorced/separated/widowed), education level (middle school or less, high school, vocational school, and college or above), and occupation (military, white-collar, professional, farmer, labourer, business, freelance, and other). Clinical information included height, weight, total cholesterol level, and hypertension status. Height and weight were used to calculate body mass index (BMI). Hypertension was defined as a blood pressure reading of 140/90 mm Hg or higher, a self-reported history of hypertension, or the use of antihypertensive medications [24].

Lifestyle factors encompassed smoking status (never, former, current), alcohol consumption (never, former, current), vegetable intake (less than half a bowl per day, less than one and a half bowls per day, one and a half bowls or more per day), and fruit intake (less than one serving per day, one serving per day, more than one serving per day). Physical activity was assessed using the metabolic equivalent of task (MET), which was calculated based on the intensity, duration, and frequency of individual physical activity. MET levels were categorised as <3.75, 3.75≤MET<7.5, 7.5≤MET<16.5, 16.5≤MET<25.5, and ≥25.5 [25].

### Data analysis

Baseline characteristics were evaluated using χ^2^ tests for categorical variables and analysis of variance (ANOVA) for continuous variables. Covariates with missing data(all missing rates <12.3%)were handled using multiple imputation by chained equations (MICE) with five imputations through the mice package in R. Hazard ratios (HRs) and 95% confidence intervals (CIs) for the associations between sleep (sleep duration and sleep disorder) and the risk of all-cause mortality across different glycaemic statuses were estimated using multivariable Cox proportional hazards regression models. Model 1 was adjusted for age and sex. Model 2 included additional adjustments for marital status, education level, and occupation. Model 3 further adjusted for BMI, total cholesterol level, hypertension, smoking status, alcohol consumption, physical activity, vegetable intake, and fruit intake.

In addition, we examined the joint association between sleep (sleep duration and sleep disorder) and glycaemic status in relation to all-cause mortality risk. For the joint association analysis, Model 1 was adjusted for age and sex. Model 2 additionally adjusted for marital status, educational attainment, and occupation, while Model 3 included further adjustments for clinical and lifestyle factors. Kaplan-Meier survival curves were plotted to visualise survival differences across glycaemic and sleep subgroups.

Stratified analyses were conducted based on sex (male or female) and age (>60 or ≤60 years) to further explore the relationship between sleep, glycaemic status, and all-cause mortality risk. Moreover, interaction terms between sleep duration and glycaemic status were included to test effect modification. Four sensitivity analyses were performed: one excluding participants whose endpoint occurred within three years, one excluding participants with cardiovascular disease or cancer at baseline, and one excluding participants with missing covariate data. In the sleep disorder questionnaire, use of sleeping pills was one of the response categories. Given that sleeping pill use may reflect underlying chronic illness or confounding by indication, we performed a sensitivity analysis by excluding this group and re-assessing the associations of other sleep disorder types (*e.g*. difficulty initiating sleep, fragmented sleep, dreamy sleep) with mortality. In addition, time varying exposure model using four observation points was also included to further validate the results. All statistical analyses were conducted using SAS software, version 9.3 (SAS Institute Inc., Cary, NC, USA) and *R*, version 4.3.3 (R Foundation for Statistical Computing, Vienna, Austria). A two-sided *P*-value of <0.05 was considered statistically significant.

## RESULTS

### Baseline characteristics of participants

Among the 534 238 participants, 363 863 had normoglycaemia with a mean age of 36.30 (standard deviation (SD) = 12.93), 144 602 were classified as having prediabetes with a mean age of 43.92 (SD = 14.13), and 25 773 had diabetes with a mean age of 55.08 (SD = 12.47) at baseline. Detailed baseline characteristics are presented in **Table 1**. A total of 370 891 participants reported sleeping between six to eight hours per night, while 214 363 reported no sleep disorder. Among those with normoglycaemia, prediabetes, and diabetes, 70.4, 68.4, and 61.3% reported sleeping 6–8 hours, respectively. Regarding sleep disorders, 40.1% of participants with normoglycaemia, 39.8% with prediabetes, and 41.5% with diabetes reported no sleep disorder. The diabetes group had the highest proportions of current drinkers and current smokers and was characterised by older age, higher physical activity levels, lower educational attainment, higher prevalence of hypertension, and higher body mass index (BMI). The absolute death rates, of people with normoglycaemia, prediabetes, and diabetes were 359.01, 680.55, and 2298.88 per 100 000 person-years, respectively.

**Table 1:** Baseline characteristics of participants by glycaemic status

Characteristic			Glycaemic status			*P-*value
	**Total (n = 534 238)**		**Normoglycaemia (n = 363 863)**	**Prediabetes (n = 144 602)**	**Diabetes (n = 25 773)**	
**Age, x̄ ± SD**		39.27 ± 14.12	36.30 ± 12.93	43.92 ± 14.13	55.08 ± 12.47	<0.001
**Gender, n (%)**						<0.001
Male		256 925 (48.09)	155 630 (42.77)	87 402 (60.44)	13 893 (53.91)	
Female		277 313 (51.91)	208 233 (57.23)	57 200 (39.56)	11 880 (46.09)	
**Absolute risk estimates (per 100 000 person-years)**		523.80	359.01	680.55	2298.88	
**Marital status, n (%)**						<0.001
Married		332 734 (62.28)	209 606 (57.61)	102 761 (71.06)	20 367 (79.02)	
Single		167 654 (31.38)	135 558 (37.26)	30 509 (21.10)	1587 (6.16)	
Divorced/separated/widowed		33 850 (6.34)	18 699 (5.14)	11 332 (7.84)	3819 (14.82)	
**Education, n (%)**						<0.001
Middle school or less		129 264 (24.20)	72 375 (19.89)	42 774 (29.58)	14 115 (54.77)	
High school		122 125 (22.86)	86 303 (23.72)	30 932 (21.39)	4890 (18.97)	
Professional school		106 685 (19.97)	78 033 (21.45)	25 722 (17.79)	2930 (11.37)	
College or above		176 164 (32.97)	127 152 (34.95)	45 174 (31.24)	3838 (14.89)	
**Occupation, n (%)**						<0.001
Military		4496 (0.84)	3371 (0.93)	1019 (0.70)	106 (0.41)	
White-collar		51 831 (9.70)	35 880 (9.86)	14 174 (9.80)	1777 (6.89)	
Profession		22 818 (4.27)	17 110 (4.70)	5118 (3.54)	590 (2.29)	
Farmer		13 741 (2.57)	7 846 (2.16)	4515 (3.12)	1380 (5.35)	
Worker	89 727 (16.80)		59 292 (16.30)	27 558 (19.06)	2877 (11.16)	
Business	148 233 (27.75)		103 422 (28.42)	39 356 (27.22)	5455 (21.17)	
Freelance	33 000 (6.18)		23 304 (6.40)	8025 (5.55)	1671 (6.48)	
Other	170 392 (31.89)		113 638 (31.23)	44 837 (31.01)	11 917 (46.24)	
**Hypertension**						<0.001
No	440 138 (82.39)		322 740 (88.70)	105 513 (72.97)	11 885 (46.11)	
Yes	94 100 (17.61)		41 123 (11.30)	39 089 (27.03)	13 888 (53.89)	
**Total cholesterol, x̄ ± SD**	192 60 ± 37.24		187.99 ± 35.39	200.91 ± 37.78	211.01 ± 45.18	<0.001
**BMI**	22.94 ± 3.73		22.22 ± 3.50	24.28 ± 3.68	25.60 ± 3.92	<0.001
**Smoke**						<0.001
Never	384 888 (72.04)		268 889 (73.90)	99 027 (68.48)	16 972 (65.85)	
Ever	116 828 (21.87)		76 553 (21.04)	34 063 (23.56)	6212 (24.10)	
Current	32 522 (6.09)		18 421 (5.06)	11 512 (7.96)	2589 (10.05)	
**Drink**						<0.001
Never	427 460 (80.01)		299 118 (82.21)	109 433 (75.68)	18 909 (73.37)	
Ever	91 640 (17.15)		56 016 (15.39)	30 343 (20.98)	5281 (20.49)	
Current	15 138 (2.83)		8729 (2.40)	4826 (3.34)	1583 (6.14)	
**Physical activity**						<0.001
MET<3.75	270 028 (50.54)		189 328 (52.03)	69 056 (47.76)	11 644 (45.18)	
3.75≤MET<7.5	131 047 (24.53)		91 410 (25.12)	34 127 (23.60)	5510 (21.38)	
7.5≤MET<16.5	90 544 (16.95)		57 818 (15.89)	27 115 (18.75)	5611 (21.77)	
16.5≤MET<25.5	26 188 (4.90)		15 596 (4.29)	8691 (6.01)	1901 (7.38)	
MET≥25.5	16 431 (3.08)		9711 (2.67)	5613 (3.88)	1107 (4.30)	
**Vegetable consumption**						<0.001
Less than half bowl per day	81 841 (15.32)		56 296 (15.47)	21 646 (14.97)	3900 (15.13)	
Less than one and half bowls per day	299 915 (56.14)		203 938 (56.05)	81 989 (56.70)	13 988 (54.27)	
One and half bowls or more per day	152 482 (28.54)		103 630 (28.48)	40 967 (28.33)	7885 (30.59)	
**Fruit consumption**						<0.001
Less than one serving per day	194 527 (36.41)		136 396 (37.49)	49 651 (34.34)	8480 (32.90)	
One serving per day	271 862 (50.89)		182 325 (50.11)	75 808 (52.43)	13 729 (53.27)	
More than one serving per day	67 849 (12.70)		45 142 (12.41)	19 143 (13.24)	3564 (13.83)	
**Sleep time**						<0.001
Less than 6 h	110 725 (20.73)		71 142 (19.55)	32 814 (22.69)	6769 (26.26)	
6–8 h	370 891(69.42)		256 213 (70.41)	98 870 (68.37)	15 808 (61.34)	
More than 8 h	52 622 (9.85)		36 508 (10.03)	12 918 (8.93)	3196 (12.40)	
**Sleep type**						<0.001
Sleep well	214 363 (40.13)		144 942 (39.83)	60 025 (41.51)	9396 (36.46)	
Difficult to fall asleep	61 475 (11.51)		40 782 (11.21)	16 797 (11.62)	3896 (15.12)	
Can fall asleep but easily awake	161 293 (30.19)		107 465 (29.53)	45 110 (31.20)	8718 (33.83)	
Dreamy sleep	90 118 (16.87)		66 760 (18.35)	20 368 (14.09)	2990 (11.60)	
Use of sleeping pills or drugs	6989 (1.31)		3914 (1.08)	2302 (1.59)	773 (3.00)	
**Sleep disorder**						<0.001
No	214 363 (40.13)		144 942 (39.83)	60 025 (41.51)	9396 (36.46)	
Yes	319 875 (59.87)		218 921 (60.17)	84 577 (58.49)	16 377 (63.54)	

MET – metabolic equivalent of task, SD – standard deviation, x̄ – mean

### Association of sleep patterns and all-cause mortality across different glycaemic statuses

**Table 2** presents the associations between sleep patterns and all-cause mortality risk across different glycaemic statuses. During a median follow-up of 19 years, a total of 52 208 participants died, yielding a mortality rate of 9.77%. Compared with participants who reported sleeping 6–8 hours, short sleep duration (<6 hours) was associated with an increased mortality risk in the normoglycaemia group (HR = 1.05; 95% CI = 1.02–1.08), while no significant associations were observed in the prediabetes or diabetes group. Sleep duration exceeding the recommended 6–8 hours was associated with increased all-cause mortality risk. Among individuals with normoglycaemia, sleeping more than 8 hours was linked to a 19% higher risk of death (Model 3: HR = 1.19; 95% CI = 1.15–1.24). For prediabetes and diabetes groups, the excess risks were 24% (Model 3: HR = 1.24; 95% CI = 1.19–1.30) and 29% (Model 3: HR = 1.29; 95% CI = 1.22–1.36), respectively.

**Table 2:** Associations between sleep duration, sleep disorder, and risks of all-cause mortality by glycaemic status

			HR (95% CI)		
**Sleep**	**Glycaemic Status**	**No.**	Model 1*	Model 2*	Model 3*
**Sleep duration**					
	Normoglycaemia				
Less than 6 h		71 142	1.07 (1.04–1.11)†	1.05 (1.02–1.08)†	1.05 (1.02–1.08)†
6–8 h		256 213	Reference	Reference	Reference
More than 8 h		36 508	1.31 (1.26–1.36)†	1.24 (1.19–1.28)†	1.19 (1.15–1.24)†
	Prediabetes				
Less than 6 h		32 814	1.04 (1.01–1.08)†	1.02 (0.99–1.06)	1.02 (0.98–1.05)
6–8 h		98 870	Reference	Reference	Reference
More than 8 h		12 918	1.36 (1.30–1.42)†	1.29 (1.23–1.35)†	1.24 (1.19–1.30)†
	Diabetes				
Less than 6 h		6769	1.00 (0.96–1.05)	0.99 (0.94–1.04)	0.98 (0.94–1.03)
6–8 h		15 808	Reference	Reference	Reference
More than 8 h		3196	1.38 (1.31–1.46)†	1.32 (1.25–1.40)†	1.29 (1.22–1.36)†
**Sleep disorder**					
	Normoglycaemia				
No		144 942	Reference	Reference	Reference
Yes		218 921	1.04 (1.02–1.07)†	1.02 (1.00–1.05)	1.02 (0.99–1.05)
	Prediabetes				
No		60 025	Reference	Reference	Reference
Yes		84 577	1.06 (1.02–1.09)†	1.04 (1.01–1.07)†	1.04 (1.01–1.07)†
	Diabetes				
No		9396	Reference	Reference	Reference
Yes		16 377	1.08 (1.04–1.13)†	1.08 (1.03–1.12)†	1.07 (1.02–1.11)†

CI – confidence interval, HR – hazard ratio

*Model 1adjusted for age and sex. Model 2 adjusted for age, sex, marriage status, occupation, education. Model 3 adjusted for age, sex, marriage status, occupation, education, hypertension, body mass index, total cholesterol, smoke, drink, physical activity, vegetable consumption, and fruit consumption

†Statistically significant at *P* < 0.05.

Sleep disorders were also found to be associated with increased mortality risk. Across Models 1 to 3, the risk of all-cause mortality rose progressively with worsening glycaemic control. Although not all associations reached statistical significance across models, the data indicated that individuals with prediabetes (Model 3: HR = 1.04; 95% CI = 1.01–1.07) and diabetes (Model 3: HR = 1.07; 95% CI = 1.02–1.11) faced elevated risks of death compared to their counterparts. To further explore the relationship between sleep disorders and all-cause mortality, we examined specific components of sleep disorders. Among the reported types, ‘difficult to fall asleep,’ ‘easily awakened after falling asleep,’ ‘dreamy sleep,’ and ‘use of sleeping pills or drugs,’ ‘difficult to fall asleep,’ and ‘use of sleeping pills or drugs’ were found to be significantly associated with increased risk of all-cause mortality across all glycaemic status groups (**Table 3**). Compared with participants who reported ‘sleeping well,’ those who reported ‘use of sleeping pills or drugs’ showed significantly higher risks of all-cause mortality: normoglycaemia group (Model 3: HR = 1.29; 95% CI = 1.19–1.39), prediabetes group (Model 3: HR = 1.32; 95% CI = 1.21–1.43), and diabetes group (Model 3: HR = 1.15; 95% CI = 1.03–1.28). In addition, reporting ‘difficult to fall asleep’ was associated with 1.08- to 1.17-fold increased risks of mortality: normoglycaemia group (Model 3: HR = 1.08; 95% CI = 1.04–1.12), prediabetes group (Model 3: HR = 1.11; 95% CI = 1.06–1.16), and diabetes group (Model 3: HR = 1.17; 95% CI = 1.11–1.28).

**Table 3:** Associations between sleep type and risks of all-cause mortality by glycaemic status

			HR (95% CI)		
**Sleep**	**Glycaemic Status**	**No.**	Model 1*	Model 2*	Model 3*
**Sleep Type**					
	Normoglycaemia				
Sleep well		144 942	Reference	Reference	Reference
Difficult to fall asleep		40 782	1.17 (1.13–1.22)†	1.11 (1.07–1.15)†	1.08 (1.04–1.12)†
Can fall asleep but easily awake		107 465	1.00 (0.97–1.03)	0.98 (0.95–1.01)	0.99 (0.96–1.02)
Dreamy sleep		66 760	0.98 (0.94–1.02)	0.98 (0.94–1.02)	0.99 (0.95–1.03)
Use of sleeping pills or drugs		3914	1.35 (1.25–1.45)†	1.35 (1.25–1.46)†	1.29 (1.19–1.39)†
	Prediabetes				
Sleep well		60 025	Reference	Reference	Reference
Difficult to fall asleep		16 797	1.20 (1.15–1.26)†	1.15 (1.10–1.20)†	1.11 (1.06–1.16)†
Can fall asleep but easily awake		45 110	1.00 (0.97–1.04)	0.99 (0.95–1.03)	1.00 (0.97–1.04)
Dreamy sleep		20 368	0.99 (0.95–1.04)	1.00 (0.95–1.05)	1.01 (0.96–1.06)
Use of sleeping pills or drugs		2302	1.36 (1.25–1.48)†	1.35 (1.24–1.47)†	1.32 (1.21–1.43)†
	Diabetes				
Sleep well		9396	Reference	Reference	Reference
Difficult to fall asleep		3896	1.24 (1.17–1.32)†	1.21 (1.14–1.28)†	1.17 (1.11–1.25)†
Can fall asleep but easily awake		8718	1.05 (1.00–1.10)	1.04 (0.99–1.10)	1.04 (0.99–1.10)
Dreamy sleep		2990	0.95 (0.89–1.02)	0.97 (0.90–1.04)	0.98 (0.91–1.05)
Use of sleeping pills or drugs		773	1.20 (1.08–1.34)†	1.22 (1.10–1.36)†	1.15 (1.03–1.28)†

CI – confidence interval, HR – hazard ratio

*Model 1 adjusted for age and sex. Model 2 adjusted for age, sex, marriage status, occupation, education. Model 3 adjusted for age, sex, marriage status, occupation, education, hypertension, body mass index, total cholesterol, smoke, drink, physical activity, vegetable consumption, and fruit consumption.

†Statistically significant at *P* < 0.05.

Kaplan-Meier survival curves stratified by glycaemic status showed consistently lower survival probabilities among individuals reporting sleep disorders compared to those without, across all glycaemic groups (Figure S1, Panels D–F in the **Online Supplementary Document**). In contrast, the patterns for sleep duration were more heterogeneous across glycaemic statuses (Figure S1, Panels A–C in the **Online Supplementary Document**). Among individuals with prediabetes and diabetes, those who reported sleeping more than eight hours exhibited lower survival probabilities compared to those sleeping less than six hours or 6–8 hours. However, in the normoglycaemia group, the lowest survival was observed in participants with short sleep duration (<6 hours), followed by long sleep (>8 hours), with the best survival seen in the 6–8-hour reference group.

### Joint effects of sleep pattern and glycaemia status on all-cause mortality

**Figure 2:**
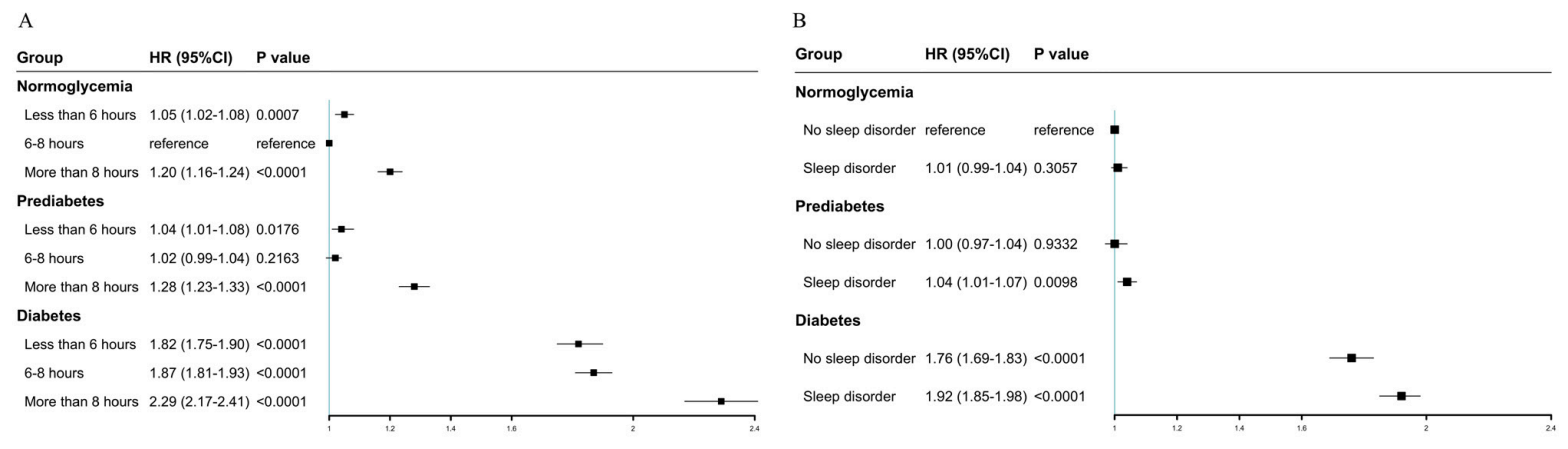
Joint analysis between sleep and glycaemic status on all-cause mortality. **Panel A.** Joint effect of sleep duration and glycaemic status on all-cause mortality. **Panel B.** Joint effect of sleep disorder and glycaemic status on all-cause mortality. CI – confidence interval, HR – hazard ratio.

The joint associations between glycaemic status and sleep duration were evaluated (**Figure 2**). Among the various combinations of glycaemic status and sleep duration, individuals with diabetes who slept more than eight hours had the highest all-cause mortality risk, with a 2.29-fold increase (Model 3: HR = 2.29; 95% CI = 2.17–2.41) (**Figure 2**). Across all sleep duration categories, individuals with diabetes exhibited the highest mortality risks. Specifically, insufficient, recommended, and excessive sleep durations were associated with 1.82-fold (Model 3: HR = 1.82; 95% CI = 1.75–1.90), 1.87-fold (Model 3: HR = 1.87; 95% CI = 1.81–1.93), and 2.29-fold (Model 3: HR = 2.29; 95% CI = 2.17–2.41) increased risks, respectively, in individuals with diabetes. Notably, prolonged sleep duration was linked to higher mortality risk compared to either insufficient or optimal sleep duration. No statistically significant joint associations between glycaemic status and sleep disorder were observed in the normoglycaemia and prediabetes groups. However, in the diabetes group, the joint effect of sleep disorder on mortality reached a 1.92-fold increase (HR = 1.92; 95% CI = 1.85–1.98).

A formal test of interaction between sleep duration and glycaemic status on all-cause mortality (Table S1 in the **Online Supplementary Document**) showed statistically significant interactions across all glycaemic groups: *P*_interaction = 5.37 × 10^−6^ for normoglycaemia group, 1.22 × 10^−12^ for prediabetes group, and 4.86 × 10^−15^ for diabetes group. In contrast, the interaction between sleep disorder and glycaemic status was not significant for normoglycaemia group (*P* = 0.3828) but reached statistical significance for prediabetes group (*P* = 0.0327) and diabetes group (*P* = 0.0053).

### Subgroup and sensitivity analyses

The findings from the stratified and sensitivity analyses were consistent with those of the main analysis. Excluding participants whose endpoints occurred within three years of their baseline visits (Table S2 in the **Online Supplementary Document**) continued to demonstrate the association between excessive sleep duration and increased all-cause mortality risk (normoglycaemia: HR = 1.19; 95% CI = 1.14–1.24; prediabetes: HR = 1.23; 95% CI = 1.18–1.29; diabetes: HR = 1.27; 95% CI = 1.20–1.35). While sleeping less than six hours was discovered to have an association with a higher risk of mortality in the normoglycaemia group (HR = 1.05; 95% CI = 1.02–1.08). Sleep disorder was also discovered to have association with higher risk of mortality in prediabetes and diabetes group (prediabetes: HR = 1.03; 95% CI = 1.00–1.07; diabetes: HR = 1.05; 95% CI = 1.01–1.10).

Similar associations were observed among participants who were free of cancer and cardiovascular disease at baseline (Table S3 in the **Online Supplementary Document**). Excessive sleep duration remained linked to elevated mortality risk in individuals with normoglycaemia group (HR = 1.16; 95% CI = 1.11–1.21), prediabetes group (HR = 1.21; 95% CI = 1.15–1.27), and diabetes group (HR = 1.26; 95% CI = 1.18–1.34). Additionally, the co-occurrence of sleep disorders and diabetes was associated with an increased risk of mortality (prediabetes: HR = 1.04; 95% CI = 1.01–1.08; diabetes: HR = 1.07; 95% CI = 1.02–1.12). In addition, sleeping less than six hours was only found to be associated with a higher risk of mortality in the normoglycaemia group (HR = 1.06; 95% CI = 1.02–1.09).

When participants with missing covariates were excluded, the results remained consistent with the main analysis (Table S4 in the **Online Supplementary Document**), showing that sleeping more than eight hours was associated with increased mortality risk in normoglycaemia group (HR = 1.19; 95% CI = 1.14–1.24), prediabetes group (HR = 1.22; 95% CI = 1.17–1.28), and diabetes (HR = 1.26; 95% CI = 1.18–1.34) and sleeping less than six hours was associated with increased mortality risk in normoglycaemia group (HR = 1.04; 95% CI = 1.01–1.08).

After excluding participants who reported use of sleeping pills (n = 6989), the association between sleep disorders and risk of mortality was discovered across all glycaemic groups (normoglycaemia: HR = 1.05; 95% CI = 1.02–1.09; prediabetes: HR = 1.09; 95% CI = 1.05–1.13; diabetes: HR = 1.09; 95% CI = 1.04–1.15) (Table S5 in the **Online Supplementary Document**). These results suggest that the observed associations were not solely driven by the sleeping pill subgroup. Time varying exposure model using four observations showed similar trends in sleep duration and all-cause mortality (Table S6 in the **Online Supplementary Document**)). In the normoglycaemia group, both longer and shorter sleep were associated with a higher risk of all-cause mortality (less than six hours of sleep: HR = 1.13; 95% CI = 1.01–1.26; more than eight hours of sleep: HR = 1.30; 95% CI = 1.09–1.55). For prediabetes and diabetes groups, more than 8 hours of sleep was associated with a higher risk of mortality (prediabetes: HR = 1.25; 95% CI = 1.05–1.50; diabetes: HR = 1.45; 95% CI = 1.19–1.78).

Age- and sex-stratified analyses also yielded similar results. Sleeping more than eight hours was associated with elevated all-cause mortality risk in both males and females (Table S7 in the **Online Supplementary Document**). However, sleep disorders showed a statistically significant association with increased mortality only among male participants with diabetes (HR = 1.24; 95% CI = 1.15–1.34). Stratified analysis by age (≥60 or <60 years) indicated that, regardless of age group, insufficient sleep (less than 6 hours) was associated with increased mortality risk among individuals with normoglycaemia (<60 years: HR = 1.21; 95% CI = 1.16–1.26; ≥60 years: HR = 1.06; 95% CI = 1.02–1.10). In contrast, among individuals with diabetes, excessive sleep (more than eight hours) was linked to higher mortality risk in both age groups (Table S8 in the **Online Supplementary Document**). Furthermore, individuals aged 60 years or younger with diabetes and sleep disorders were found to have an elevated risk of all-cause mortality (HR = 1.11; 95% CI = 1.03–1.18).

## DISCUSSION

Based on a prospective cohort of 534 238 individuals from an Asian population, we found that prolonged sleep duration and the presence of sleep disorders were associated with higher individual risks of all-cause mortality among people with prediabetes and diabetes. Specifically, sleeping more than eight hours was linked to an increased risk of all-cause mortality across all glycaemic statuses when compared to the optimal sleep duration of six to eight hours. In addition, for those without prediabetes and diabetes, sleeping less than six hours was associated with higher risk of mortality. In contrast, sleep disorders were associated with higher mortality risk in individuals with prediabetes and diabetes, but not in those with normoglycaemia. Furthermore, when compared to individuals with normoglycaemia who slept six to eight hours, those with diabetes who slept more than eight hours exhibited the highest risk of all-cause mortality.

Although sleep plays a substantial role in the incidence and progression of diabetes as well as mortality, research examining the interplay between sleep, glycaemic status, and mortality remains limited, particularly in Asian populations. A few cohort studies have investigated the relationship between sleep duration and all-cause mortality in diabetic individuals, and their findings are consistent with ours [26–29]. For example, Wang et al. found that among 273 029 participants in the US National Health Interview Survey, the association between sleep duration and mortality risk differed between individuals with and without diabetes, with extremely long sleep durations being significantly linked to increased all-cause mortality among those with diabetes [27]. Similarly, Inoue et al. reported that longer sleep duration was associated with higher all-cause mortality risk, even more so than shorter sleep duration [28]. However, these studies predominantly focused on non-Asian populations and exclusively examined diabetes. Research involving Asian populations, such as a study of 12 526 Asian diabetic patients [29] and another with 51 603 Korean participants [30], also supported a J-shaped relationship between sleep duration and mortality risk. Our study expands upon these findings by incorporating prediabetes as a distinct glycaemic status and demonstrating that both insufficient and prolonged sleep were associated with elevated all-cause mortality risks in this group. This highlights the importance of considering the full spectrum of glucose regulation when evaluating the health impact of sleep behaviours.

In studies on sleep disorders, current findings in the literature remain inconsistent and inconclusive. For example, von Schantz M et al. reported that diabetes, when accompanied by frequent sleep disturbances, was associated with a significantly higher individual risk of mortality [31] (HR = 1.87; 95% CI = 1.75–2.01). Another cohort study found that diabetes doubled the risk of mortality regardless of the presence of a sleep disorder, although the risk was higher in those without a diagnosed sleep disorder [28]. These discrepancies may stem from the heterogeneity in how sleep disorders are identified – some studies relied on self-reported data, while others included only physician-diagnosed cases. Additionally, the varying operational definitions of sleep disorder across studies may influence the results. Definitions have ranged from ‘difficulty falling asleep at night and frequent nighttime awakenings’ to broader combinations of symptoms. Our findings were consistent with those of Schantz et al. [25], supporting the notion that sleep disorders play a significant role in all-cause mortality among individuals with diabetes.

Sleep is a complex behaviour, and recent research [20,29] has increasingly adopted multi-dimensional frameworks to define it. A recent UK Biobank study involving 12 770 individuals found that healthy sleep patterns were associated with lower all-cause mortality risk among diabetic patients [20]. While studying sleep through a single lens, such as sleep duration, can yield limited insights, combining multiple sleep behaviours into a composite indicator may obscure the contributions of individual components. Our study offered a more comprehensive and detailed definition of sleep by analysing multiple dimensions of sleep disorders, including the use of sleeping pills or drugs, difficulty falling asleep, dreamy sleep, and the ability to fall asleep but waking easily, both individually and collectively, in addition to sleep duration. The associations between long sleep duration and mortality may be partially explained by underlying comorbidities, such as frailty, depression, or subclinical diseases that increase sleep need and also elevate mortality risk. The use of sleep medications may reflect more severe insomnia or psychiatric conditions, rather than a direct pharmacological effect, which warrants cautious interpretation.

To our knowledge, this is the largest cohort study conducted in an Asian population that comprehensively examined sleep from multiple dimensions and differentiated glucose intolerance into prediabetes and diabetes groups, thereby filling the gap in understanding the association between sleep and all-cause mortality in individuals with prediabetes. Our study provided new evidence showing that not only individuals with diabetes, but also those with prediabetes, faced greater mortality risks when experiencing prolonged sleep. These findings emphasise that identical sleep behaviours can have different impacts on mortality depending on an individual’s glycaemic status.

Potential mechanisms may involve inflammation and diabetic coma. Sleep disruptions have been associated with endocrine disorders [32], impaired immune function, increased oxidative stress [33], elevated inflammatory responses, and endothelial dysfunction [34]. A meta-analysis [35] reported that disrupted sleep patterns were linked to higher levels of C-reactive protein (CRP) and interleukin-6 (IL-6). While shorter sleep duration was correlated with increased CRP levels, excessively prolonged sleep was associated with elevations in both CRP and IL-6. Another possible mechanism involves diabetic coma. Both hyperglycaemia and hypoglycaemia can lead to loss of consciousness and, if left untreated, may compromise brain function or be life-threatening [36]. Additionally, the autonomic nervous system plays a critical role in regulating many essential physiological functions during sleep [37]. Sleep disturbances may induce autonomic dysfunction [38], which can further impair metabolic regulation and exacerbate conditions such as obesity [39], impaired glucose tolerance [40], and insulin resistance in individuals with impaired glucose metabolism.

However, there are several limitations to this study. First, sleep-related information was collected through self-reported questionnaires, which may be subject to recall bias and measurement error. Participants, particularly short sleepers, may underestimate their actual sleep duration. While this bias is likely non-differential and may attenuate the associations [41,42], it may still reduce measurement precision. In addition, when collecting the sleep duration data, it was treated as a general category, thus not providing data for more detailed analysis. Nevertheless, this instrument has been applied in previous peer-reviewed studies based on the same cohort [43].

Second, although sleep patterns may fluctuate over time due to aging, lifestyle, health changes, cultural differences, environmental differences, and health systems, only baseline sleep data were available for the majority of participants. To address this, we conducted a sensitivity analysis using time varying exposure model from participants with multiple follow-up visits, and the associations remained largely consistent. These findings suggest relative stability in the observed sleep–mortality associations. Third, due to the observational nature of the study, causality cannot be established. Although extensive covariates were adjusted for, residual confounding (*e.g*. depressive symptoms or medication adherence) may remain. Finally, although we cannot entirely rule out the possibility of reverse causation, several factors reduce this concern: the prospective cohort design ensured that sleep information was collected prior to the occurrence of death, which inherently lowers the likelihood of reverse causation. In addition, we conducted sensitivity analyses excluding participants who died within the first three years of follow-up and those with cardiovascular diseases and cancer at baseline. The associations remained largely consistent, suggesting that pre-existing subclinical illness is unlikely to fully account for the observed relationships. Nevertheless, we acknowledge that undiagnosed or progressive conditions (*e.g*. heart failure, cancer, or frailty) may still influence sleep behaviours and subsequent mortality risk, and this limitation should be considered when interpreting the findings. Therefore, further research is required to clarify the mechanisms and causal relationships between sleep behaviours and all-cause mortality among individuals with varying glycaemic statuses.

## CONCLUSIONS

This study examined the association between sleep and all-cause mortality across different glycaemic statuses. The findings indicated that the effects of sleep disorders, as well as insufficient or prolonged sleep duration, on all-cause mortality risk vary according to glycaemic status. While suboptimal sleep duration was associated with all-cause mortality among individuals with normoglycaemia, prolonged sleep duration was the primary risk factor for those with impaired glucose regulation. Furthermore, an association between sleep disorders and all-cause mortality was observed specifically in patients with diabetes. These findings highlight the need to consider glycaemic status when evaluating sleep-related health risks. While our results support the potential value of sleep health in chronic disease management, they should be interpreted with caution given the observational design. Further longitudinal and interventional studies are warranted to assess whether improving sleep can reduce mortality risk in at-risk populations.

## Additional material


Online Supplementary Document
