# Supplementary material for: Association of sleep and glycaemic status with all-cause mortality: a prospective cohort study
Source: J Glob Health. 2026 Jan 12;16:04002. doi: 10.7189/jogh.16.04002 (PMC12793928; doi:10.7189/jogh.16.04002)

**Supplementary Table 1 Interaction between Sleep and Glycemic Status on Mortality**

| Sleep Exposure | Glycemic Status | P for Interaction |
|---|---|---|
| **Sleep Duration** | | |
| | Normoglycemia | $5.37 \times 10^{-6}$ |
| | Prediabetes | $1.22 \times 10^{-12}$ |
| | Diabetes | $4.86 \times 10^{-15}$ |
| **Sleep Disorder** | | |
| | Normoglycemia | 0.3828 |
| | Prediabetes | 0.0327 |
| | Diabetes | 0.0053 |
| **Sleep Type** | | |
| | Normoglycemia | 0.8254 |
| | Prediabetes | 0.1194 |
| | Diabetes | 0.5484 |

**Supplementary Table 2 Association between sleep and all-cause mortality by glycemic status excluding participants whose endpoint occurred within 3 years of baseline visit**

| Glycemic status | Sleep | HR (95%CI) | | |
|---|---|---|---|---|
| | | **Model 1** | **Model 2** | **Model 3** |
| | **Sleep Duration** | | | |
| Normoglycemia | | | | |
| | Less than 6 hours | **1.07 (1.04-1.11)** | **1.05 (1.02-1.08)** | **1.05 (1.02-1.08)** |
| | 6-8 hours | reference | reference | reference |
| | More than 8 hours | **1.30 (1.25-1.35)** | **1.23 (1.19-1.28)** | **1.19 (1.14-1.24)** |
| Prediabetes | | | | |
| | Less than 6 hours | **1.04 (1.00-1.08)** | 1.02 (0.98-1.06) | 1.01 (0.98-1.05) |
| | 6-8 hours | reference | reference | reference |
| | More than 8 hours | **1.34 (1.28-1.40)** | **1.28 (1.22-1.34)** | **1.23 (1.18-1.29)** |
| Diabetes | | | | |
| | Less than 6 hours | 1.00 (0.95-1.05) | 0.98 (0.94-1.03) | 0.98 (0.93-1.03) |
| | 6-8 hours | reference | reference | reference |
| | More than 8 hours | **1.36 (1.28-1.44)** | **1.30 (1.23-1.38)** | **1.27 (1.20-1.35)** |
| | **Sleep Disorder** | | | |
| Normoglycemia | | | | |
| | No | reference | reference | reference |
| | Yes | **1.04 (1.01-1.06)** | 1.02 (0.99-1.04) | 1.01 (0.99-1.04) |
| Prediabetes | | | | |
| | No | reference | reference | reference |
| | Yes | **1.05 (1.01-1.08)** | 1.03 (1.00-1.07) | **1.03 (1.00-1.07)** |
| Diabetes | | | | |
| | No | reference | reference | reference |
| | Yes | **1.06 (1.02-1.11)** | **1.06 (1.01-1.11)** | **1.05 (1.01-1.10)** |
| | **Sleep Type** | | | |
| Normoglycemia | | | | |

| | | | | |
|---|---|---|---|---|
| | Sleep well | reference | reference | reference |
| | Difficult to fall asleep | **1.15 (1.11-1.20)** | **1.09 (1.05-1.13)** | **1.06 (1.02-1.10)** |
| | Can fall asleep but easily awake | 1.00 (0.97-1.03) | 0.98 (0.95-1.01) | 0.99 (0.96-1.02) |
| | Dreamy sleep | 0.98 (0.94-1.02) | 0.98 (0.94-1.02) | 0.98 (0.94-1.03) |
| | Use of sleeping pills or drugs | **1.34 (1.24-1.45)** | **1.34 (1.24-1.45)** | **1.29 (1.19-1.39)** |
| Prediabetes | | | | |
| | Sleep well | reference | reference | reference |
| | Difficult to fall asleep | **1.18 (1.12-1.23)** | **1.12 (1.07-1.18)** | **1.09 (1.04-1.15)** |
| | Can fall asleep but easily awake | 1.00 (0.96-1.03) | 0.99 (0.95-1.02) | 1.00 (0.96-1.04) |
| | Dreamy sleep | 1.00 (0.95-1.05) | 1.01 (0.96-1.06) | 1.02 (0.97-1.07) |
| | Use of sleeping pills or drugs | **1.33 (1.22-1.46)** | **1.33 (1.22-1.45)** | **1.30 (1.19-1.42)** |
| Diabetes | | | | |
| | Sleep well | reference | reference | reference |
| | Difficult to fall asleep | **1.22 (1.15-1.30)** | **1.18 (1.11-1.26)** | **1.15 (1.08-1.23)** |
| | Can fall asleep but easily awake | 1.03 (0.98-1.08) | 1.02 (0.97-1.08) | 1.02 (0.97-1.08) |
| | Dreamy sleep | 0.96 (0.89-1.03) | 0.97 (0.91-1.05) | 0.99 (0.92-1.06) |
| | Use of sleeping pills or drugs | **1.20 (1.07-1.34)** | **1.22 (1.09-1.36)** | **1.15 (1.03-1.29)** |

Note: excluding participants whose endpoint occurred within 3 years of baseline visit

Model 1: adjusted for age and sex. Model 2: adjusted for age, sex, marriage status, occupation, education.
Model 3: adjusted for age, sex, marriage status, occupation, education, hypertension, BMI, total cholesterol, smoke, drink, physical activity, vegetable consumption, and fruit consumption

**Supplementary Table 3 Association between sleep and all-cause mortality by glycemic status excluding participants with cancer and cardiovascular disease at baseline**

| | | HR (95%CI) | | |
|---|---|---|---|---|
| **Glycemic Status** | **Sleep** | **Model 1** | **Model 2** | **Model 3** |
| | **Sleep Duration** | | | |
| Normoglycemia | | **1.08 (1.05-1.12)** | **1.06 (1.02-1.09)** | **1.06 (1.02-1.09)** |
| | Less than 6 hours | reference | reference | reference |
| | 6-8 hours | **1.28 (1.23-1.33)** | **1.21 (1.16-1.26)** | **1.16 (1.11-1.21)** |
| | More than 8 hours | | | |
| Prediabetes | | | | |
| | Less than 6 hours | **1.05 (1.01-1.09)** | 1.03 (0.99-1.07) | 1.02 (0.98-1.06) |
| | 6-8 hours | reference | reference | reference |
| | More than 8 hours | **1.34 (1.27-1.40)** | **1.27 (1.21-1.33)** | **1.21 (1.15-1.27)** |
| Diabetes | | | | |
| | Less than 6 hours | 1.00 (0.95-1.06) | 0.99 (0.94-1.04) | 0.98 (0.93-1.04) |
| | 6-8 hours | reference | reference | reference |
| | More than 8 hours | **1.35 (1.27-1.44)** | **1.29 (1.21-1.37)** | **1.26 (1.18-1.34)** |
| | **Sleep Disorder** | | | |
| Normoglycemia | | | | |
| | No | reference | reference | reference |
| | Yes | **1.04 (1.01-1.07)** | 1.02 (0.99-1.05) | 1.02 (0.99-1.05) |
| Prediabetes | | | | |
| | No | reference | reference | reference |
| | Yes | **1.06 (1.02-1.09)** | **1.04 (1.00-1.08)** | **1.04 (1.01-1.08)** |
| Diabetes | | | | |
| | No | reference | reference | reference |
| | Yes | **1.08 (1.03-1.14)** | **1.08 (1.03-1.13)** | **1.07 (1.02-1.12)** |
| | **Sleep Type** | | | |
| Normoglycemia | | | | |

| | | | | |
|---|---|---|---|---|
| | Sleep well | reference | reference | reference |
| | Difficult to fall asleep | **1.18 (1.13-1.23)** | **1.12 (1.07-1.16)** | **1.08 (1.04-1.13)** |
| | Can fall asleep but easily awake | 1.00 (0.96-1.03) | 0.98 (0.95-1.01) | 0.99 (0.96-1.02) |
| | Dreamy sleep | 0.98 (0.94-1.03) | 0.98 (0.94-1.03) | 0.99 (0.95-1.03) |
| | Use of sleeping pills or drugs | **1.42 (1.30-1.55)** | **1.40 (1.28-1.53)** | **1.35 (1.24-1.48)** |
| Prediabetes | | | | |
| | Sleep well | reference | reference | reference |
| | Difficult to fall asleep | **1.21 (1.15-1.27)** | **1.15 (1.09-1.21)** | **1.12 (1.06-1.18)** |
| | Can fall asleep but easily awake | 1.01 (0.97-1.05) | 0.99 (0.96-1.03) | 1.01 (0.97-1.05) |
| | Dreamy sleep | 0.99 (0.93-1.04) | 0.99 (0.94-1.05) | 1.00 (0.95-1.06) |
| | Use of sleeping pills or drugs | **1.38 (1.25-1.53)** | **1.38 (1.25-1.53)** | **1.36 (1.23-1.51)** |
| Diabetes | | | | |
| | Sleep well | reference | reference | reference |
| | Difficult to fall asleep | **1.25 (1.17-1.34)** | **1.21 (1.13-1.30)** | **1.18 (1.11-1.27)** |
| | Can fall asleep but easily awake | 1.04 (0.99-1.10) | 1.04 (0.98-1.10) | 1.04 (0.98-1.10) |
| | Dreamy sleep | 0.96 (0.89-1.04) | 0.98 (0.90-1.06) | 0.99 (0.91-1.08) |
| | Use of sleeping pills or drugs | **1.26 (1.10-1.44)** | **1.27 (1.10-1.45)** | **1.18 (1.03-1.36)** |

Note: excluding participants with cancer and cardiovascular disease at baseline

Model 1: was adjusted for age and sex. Model 2: adjusted for age, sex, marriage status, occupation, education.
Model 3: adjusted for age, sex, marriage status, occupation, education, hypertension, BMI, total cholesterol, smoke, drink, physical activity, vegetable consumption, and fruit consumption

**Supplementary Table 4 Association between sleep and all-cause mortality by glycemic status excluding participants with missing covariates**

| | | HR (95%CI) | | |
|---|---|---|---|---|
| **Glycemic Status** | **Sleep** | **Model 1** | **Model 2** | **Model 3** |
| | **Sleep Duration** | | | |
| Normoglycemia | | **1.07 (1.04-1.11)** | **1.04 (1.01-1.08)** | **1.04 (1.01-1.08)** |
| | Less than 6 hours | reference | reference | reference |
| | 6-8 hours | **1.31 (1.26-1.37)** | **1.24 (1.19-1.29)** | **1.19 (1.14-1.24)** |
| | More than 8 hours | | | |
| Prediabetes | | | | |
| | Less than 6 hours | **1.04 (1.00-1.08)** | 1.02 (0.98-1.06) | 1.02 (0.98-1.06) |
| | 6-8 hours | reference | reference | reference |
| | More than 8 hours | **1.34 (1.28-1.41)** | **1.27 (1.21-1.34)** | **1.22 (1.17-1.28)** |
| Diabetes | | | | |
| | Less than 6 hours | 0.99 (0.94-1.05) | 0.98 (0.93-1.03) | 0.98 (0.93-1.03) |
| | 6-8 hours | reference | reference | reference |
| | More than 8 hours | **1.36 (1.27-1.44)** | **1.30 (1.22-1.38)** | **1.26 (1.18-1.34)** |
| | **Sleep Disorder** | | | |
| Normoglycemia | | | | |
| | No | reference | reference | reference |
| | Yes | **1.04 (1.01-1.07)** | 1.02 (0.99-1.05) | 1.01 (0.98-1.04) |
| Prediabetes | | | | |
| | No | reference | reference | reference |
| | Yes | **1.06 (1.02-1.09)** | **1.04 (1.01-1.08)** | **1.05 (1.01-1.08)** |
| Diabetes | | | | |
| | No | reference | reference | reference |
| | Yes | **1.08 (1.03-1.13)** | **1.08 (1.03-1.13)** | **1.07 (1.02-1.12)** |
| | **Sleep Type** | | | |
| Normoglycemia | | | | |

| | | | | |
|---|---|---|---|---|
| | Sleep well | reference | reference | reference |
| | Difficult to fall asleep | **1.17 (1.12-1.22)** | **1.10 (1.05-1.15)** | **1.06 (1.02-1.11)** |
| | Can fall asleep but easily awake | 0.99 (0.96-1.03) | 0.98 (0.94-1.01) | 0.98 (0.95-1.02) |
| | Dreamy sleep | 0.98 (0.93-1.02) | 0.98 (0.94-1.02) | 0.98 (0.94-1.03) |
| | Use of sleeping pills or drugs | **1.35 (1.24-1.48)** | **1.36 (1.25-1.48)** | **1.29 (1.18-1.40)** |
| Prediabetes | | | | |
| | Sleep well | reference | reference | reference |
| | Difficult to fall asleep | **1.19 (1.13-1.26)** | **1.14 (1.08-1.20)** | **1.10 (1.04-1.16)** |
| | Can fall asleep but easily awake | 1.01 (0.97-1.05) | 1.00 (0.96-1.04) | 1.02 (0.98-1.06) |
| | Dreamy sleep | 1.00 (0.95-1.06) | 1.01 (0.96-1.07) | 1.02 (0.97-1.08) |
| | Use of sleeping pills or drugs | **1.34 (1.22-1.48)** | **1.33 (1.20-1.46)** | **1.29 (1.17-1.42)** |
| Diabetes | | | | |
| | Sleep well | reference | reference | reference |
| | Difficult to fall asleep | **1.25 (1.16-1.33)** | **1.21 (1.13-1.29)** | **1.18 (1.10-1.26)** |
| | Can fall asleep but easily awake | 1.05 (0.99-1.11) | 1.05 (0.99-1.10) | 1.05 (0.99-1.11) |
| | Dreamy sleep | 0.95 (0.88-1.03) | 0.97 (0.90-1.05) | 0.98 (0.91-1.07) |
| | Use of sleeping pills or drugs | **1.24 (1.10-1.40)** | **1.24 (1.10-1.40)** | **1.16 (1.02-1.31)** |

Note: excluding participants with missing covariates

Model 1: was adjusted for age and sex. Model 2: adjusted for age, sex, marriage status, occupation, education.
Model 3: adjusted for age, sex, marriage status, occupation, education, hypertension, BMI, total cholesterol, smoke, drink, physical activity, vegetable consumption, and fruit consumption

**Supplementary Table 5 Association between sleep and all-cause mortality by glycemic status excluding participants using sleeping pills and drugs**

| | | HR (95%CI) | | |
|---|---|---|---|---|
| **Glycemic Status** | **Sleep** | **Model 1** | **Model 2** | **Model 3** |
| | **Sleep Disorder** | | | |
| Normoglycemia | | | | |
| | No | reference | reference | reference |
| | Yes | **1.03 (1.00 -1.06)** | 1.01 (0.98 -1.04) | 1.01 (0.98 -1.04) |
| Prediabetes | | | | |
| | No | reference | reference | reference |
| | Yes | **1.04 (1.01 -1.08)** | 1.03 (0.99 -1.06) | 1.03 (1.00 -1.06) |
| Diabetes | | | | |
| | No | reference | reference | reference |
| | Yes | **1.07 (1.03 -1.12)** | **1.07 (1.02 -1.12)** | **1.06 (1.02 -1.11)** |
| | **Sleep Type** | | | |
| Normoglycemia | | | | |
| | Sleep well | reference | reference | reference |
| | Difficult to fall asleep | **1.17 (1.13 -1.21)** | **1.11 (1.07 -1.15)** | **1.08 (1.04 -1.12)** |
| | Can fall asleep but easily awake | 1.00 (0.97 -1.03) | 0.98 (0.95 -1.01) | 0.99 (0.96 -1.02) |
| | Dreamy sleep | 0.98 (0.94 -1.02) | 0.98 (0.94 -1.02) | 0.99 (0.95 -1.03) |
| Prediabetes | | | | |
| | Sleep well | reference | reference | reference |
| | Difficult to fall asleep | **1.20 (1.15 -1.26)** | **1.14 (1.09 -1.20)** | **1.11 (1.06 -1.16)** |
| | Can fall asleep but easily awake | 1.00 (0.97 -1.04) | 0.99 (0.96 -1.03) | 1.00 (0.97 -1.04) |
| | Dreamy sleep | 0.99 (0.95 -1.04) | 1.00 (0.95 -1.05) | 1.01 (0.96 -1.06) |
| Diabetes | | | | |

| | | | |
|---|---|---|---|
| Sleep well | reference | reference | reference |
| Difficult to fall asleep | **1.25 (1.17 -1.32)** | **1.21 (1.14 -1.28)** | **1.18 (1.11 -1.25)** |
| Can fall asleep but easily awake | 1.05 (1.00 -1.10) | 1.05 (1.00 -1.10) | 1.04 (0.99 -1.10) |
| Dreamy sleep | 0.95 (0.89 -1.02) | 0.97 (0.90 -1.04) | 0.98 (0.91 -1.05) |

Note: excluding participants using sleeping pills and drugs at baseline

Model 1: was adjusted for age and sex. Model 2: adjusted for age, sex, marriage status, occupation, education.
Model 3: adjusted for age, sex, marriage status, occupation, education, hypertension, BMI, total cholesterol, smoke, drink, physical activity, vegetable consumption, and fruit consumption

**Supplementary Table 6 Association between sleep and all-cause mortality by glycemic status using time varying exposure model with 4 observations**

| Glycemic Status | Sleep | HR (95%CI) | P value |
|---|---|---|---|
| | **Sleep Duration** | | |
| Normoglycemia | | | |
| | Less than 6 hours | **1.13 (1.01-1.26)** | 0.0287 |
| | 6-8 hours | reference | |
| | More than 8 hours | **1.30 (1.09-1.55)** | 0.0042 |
| Prediabetes | | | |
| | Less than 6 hours | 0.95 (0.85-1.07) | 0.3914 |
| | 6-8 hours | reference | |
| | More than 8 hours | **1.25 (1.05-1.50)** | 0.0136 |
| Diabetes | | | |
| | Less than 6 hours | 1.04 (0.89-1.21) | 0.6555 |
| | 6-8 hours | reference | |
| | More than 8 hours | **1.45 (1.19-1.78)** | 0.0003 |
| | **Sleep Disorder** | | |
| Normoglycemia | | | |
| | No | reference | |
| | Yes | **1.18 (1.06-1.30)** | 0.0019 |
| Prediabetes | | | |
| | No | reference | |
| | Yes | 1.04 (0.94-1.15) | 0.4246 |
| Diabetes | | | |
| | No | reference | |
| | Yes | **1.15 (1.00-1.32)** | 0.0476 |

Note: Model adjusted for age, sex, marriage status, occupation, education, hypertension, BMI, total cholesterol, smoke, drink, physical activity, vegetable consumption, and fruit consumption

**Supplementary Table 7 Stratification analysis based on gender**

| | | Model 1 | | Model 2 | |
|---|---|---|---|---|---|
| **Glycemic status** | **Sleep time** | Male | Female | Male | Female |
| Normoglycemia | | | | | |
| | Less than 6 hours | **1.07 (1.03-1.11)** | 1.03 (0.99-1.07) | **1.06 (1.02-1.10)** | 1.03 (0.99-1.08) |
| | 6-8 hours | reference | reference | reference | reference |
| | More than 8 hours | **1.24 (1.19-1.31)** | **1.23 (1.16-1.30)** | **1.19 (1.13-1.25)** | **1.20 (1.13-1.27)** |
| Prediabetes | | | | | |
| | Less than 6 hours | 1.04 (1.00-1.09) | 1.00 (0.95-1.05) | 1.03 (0.99-1.08) | 0.99 (0.94-1.05) |
| | 6-8 hours | reference | reference | reference | reference |
| | More than 8 hours | **1.29 (1.22-1.36)** | **1.30 (1.21-1.40)** | **1.24 (1.17-1.31)** | **1.26 (1.17-1.35)** |
| Diabetes | | | | | |
| | Less than 6 hours | 0.98 (0.91-1.04) | 1.00 (0.94-1.07) | 0.97 (0.90-1.03) | 1.00 (0.94-1.07) |
| | 6-8 hours | reference | reference | reference | reference |
| | More than 8 hours | **1.28 (1.19-1.38)** | **1.38 (1.27-1.50)** | **1.24 (1.15-1.34)** | **1.36 (1.26-1.48)** |
| **Glycemic status** | **Sleep disorder** | | | | |
| Normoglycemia | | | | | |
| | No | reference | reference | reference | reference |
| | Yes | 0.99 (0.96-1.03) | **1.07 (1.02-1.11)** | 0.99 (0.96-1.03) | **1.07 (1.02-1.11)** |
| Prediabetes | | | | | |
| | No | reference | reference | reference | reference |
| | Yes | **1.04 (1.00-1.09)** | 1.03 (0.98-1.09) | **1.05 (1.01-1.09)** | 1.03 (0.98-1.09) |
| Diabetes | | | | | |
| | No | reference | reference | reference | reference |
| | Yes | **1.08 (1.02-1.14)** | **1.07 (1.00-1.15)** | **1.07 (1.01-1.13)** | 1.06 (0.99-1.14) |

Note: Model 1: adjusted for age, sex, marriage status, occupation, education. Model 2: adjusted for age, sex, marriage status, occupation, education, hypertension, BMI, total cholesterol, smoke, drink, physical activity, vegetable consumption, and fruit consumption

**Supplementary Table 8 Stratification analysis based on age**

| | | Model 1 | | Model 2 | |
|---|---|---|---|---|---|
| **Glycemic status** | **Sleep time** | <=60 | >60 | <=60 | >60 |
| Normoglycemia | | | | | |
| | Less than 6 hours | **1.27 (1.22-1.32)** | **1.06 (1.02-1.11)** | **1.21 (1.16-1.26)** | **1.06 (1.02-1.10)** |
| | 6-8 hours | reference | reference | reference | reference |
| | More than 8 hours | **1.06 (1.01-1.12)** | **1.30 (1.24-1.37)** | **1.09 (1.03-1.15)** | **1.25 (1.19-1.32)** |
| Prediabetes | | | | | |
| | Less than 6 hours | **1.21 (1.14-1.27)** | 1.04 (0.99-1.09) | **1.16 (1.10-1.22)** | 1.03 (0.98-1.08) |
| | 6-8 hours | reference | reference | reference | reference |
| | More than 8 hours | **1.23 (1.15-1.33)** | **1.37 (1.30-1.45)** | **1.24 (1.15-1.33)** | **1.33 (1.26-1.40)** |
| Diabetes | | | | | |
| | Less than 6 hours | 1.04 (0.96-1.12) | 1.02 (0.96-1.08) | 1.04 (0.96-1.12) | 1.01 (0.95-1.07) |
| | 6-8 hours | reference | reference | reference | reference |
| | More than 8 hours | **1.23 (1.12-1.35)** | **1.47 (1.37-1.58)** | **1.21 (1.10-1.33)** | **1.44 (1.34-1.54)** |
| **Glycemic status** | **Sleep disorder** | | | | |
| Normoglycemia | | | | | |
| | No | reference | reference | reference | reference |
| | Yes | **1.06 (1.03-1.10)** | **1.04 (1.00-1.08)** | **1.06 (1.02-1.10)** | 1.03 (0.99-1.07) |
| Prediabetes | | | | | |
| | No | reference | reference | reference | reference |
| | Yes | **1.09 (1.04-1.15)** | 1.03 (0.99-1.08) | **1.08 (1.03-1.14)** | 1.02 (0.98-1.06) |
| Diabetes | | | | | |
| | No | reference | reference | reference | reference |
| | Yes | **1.13 (1.05-1.20)** | 1.05 (0.99-1.11) | **1.11 (1.03-1.18)** | 1.03 (0.98-1.09) |

Note: Model 1: adjusted for age, sex, marriage status, occupation, education. Model 2: adjusted for age, sex, marriage status, occupation, education, hypertension, BMI, total cholesterol, smoke, drink, physical activity, vegetable consumption, and fruit consumption

**Supplementary Figure 1 Kaplan–Meier survival curves stratified by glycemic status**

## a) Normoglycemia – Sleep Duration Category

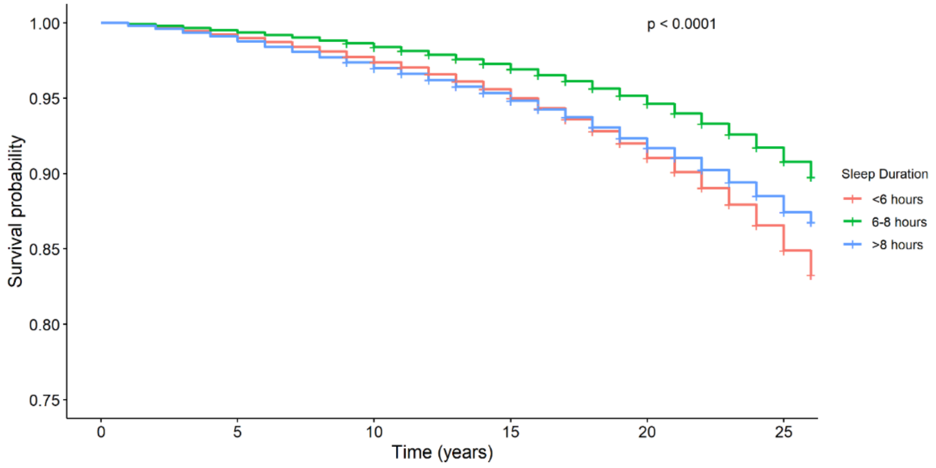


## b) Prediabetes – Sleep Duration Category

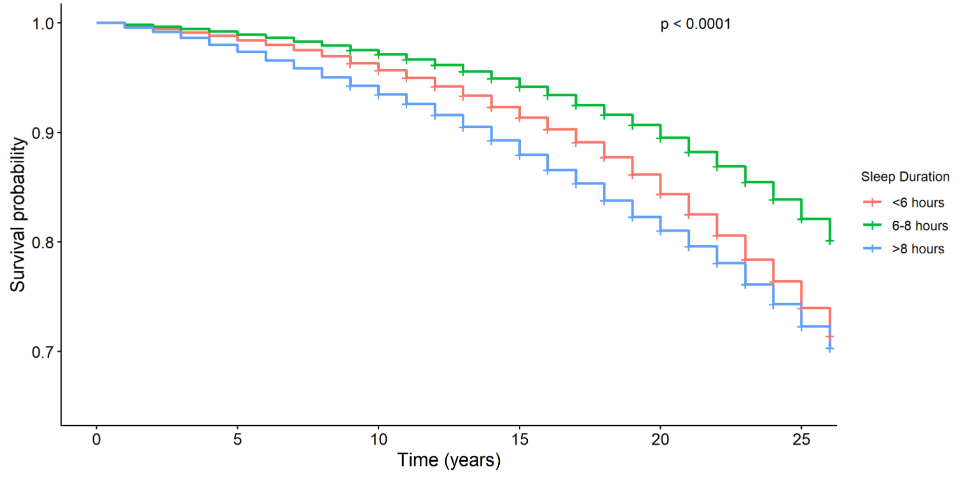

## c) Diabetes – Sleep Duration Category

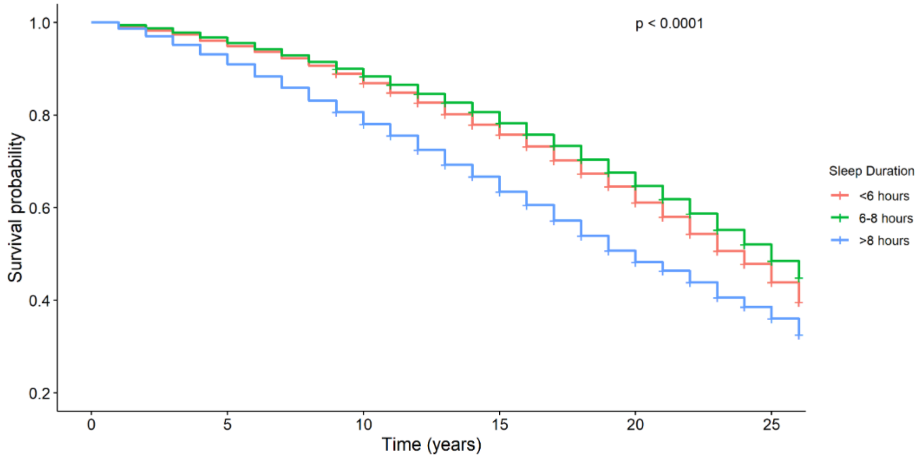


## d) Normoglycemia – Sleep Disorder Category

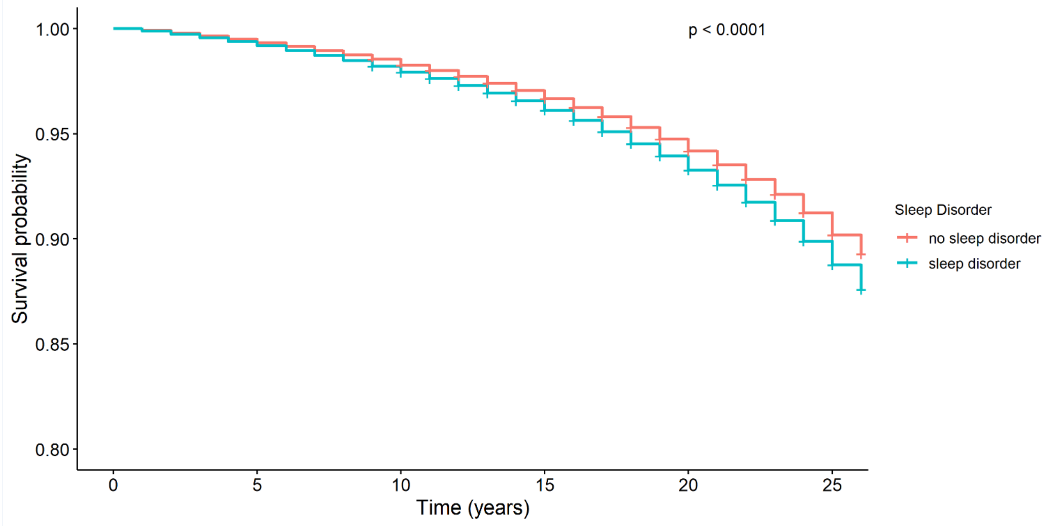

## e) Prediabetes – Sleep Disorder Category

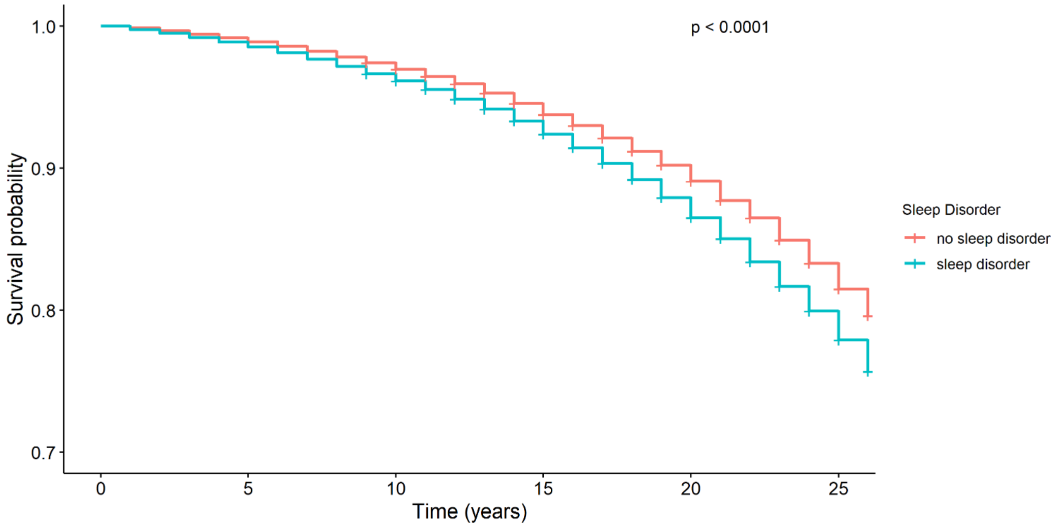


## f) Diabetes – Sleep Disorder Category

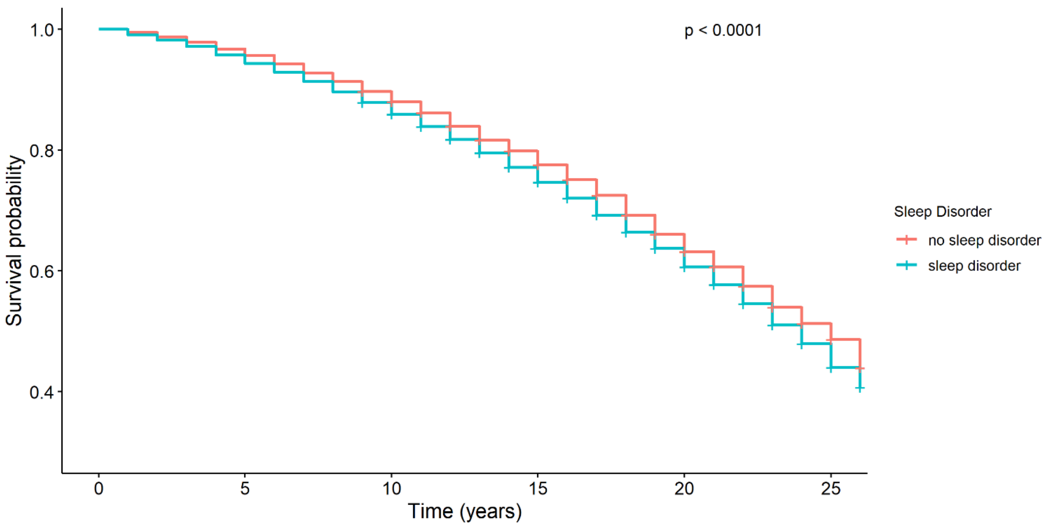

**Supplementary Figure 2** Joint association between sleep duration and glycemic status on all-cause mortality excluding participants whose endpoint was within 3 years

a) Joint effect of sleep duration and glycemic status on all-cause mortality

| Group | HR (95%CI) | P value |
|---|---|---|
| **Normoglycemia** | | |
| Less than 6 hours | 1.05 (1.02-1.08) | 0.0007 |
| 6-8 hours | reference | reference |
| More than 8 hours | 1.20 (1.16-1.24) | <0.0001 |
| **Prediabetes** | | |
| Less than 6 hours | 1.04 (1.01-1.08) | 0.0176 |
| 6-8 hours | 1.02 (0.99-1.04) | 0.2163 |
| More than 8 hours | 1.28 (1.23-1.33) | <0.0001 |
| **Diabetes** | | |
| Less than 6 hours | 1.82 (1.75-1.90) | <0.0001 |
| 6-8 hours | 1.87 (1.81-1.93) | <0.0001 |
| More than 8 hours | 2.29 (2.17-2.41) | <0.0001 |

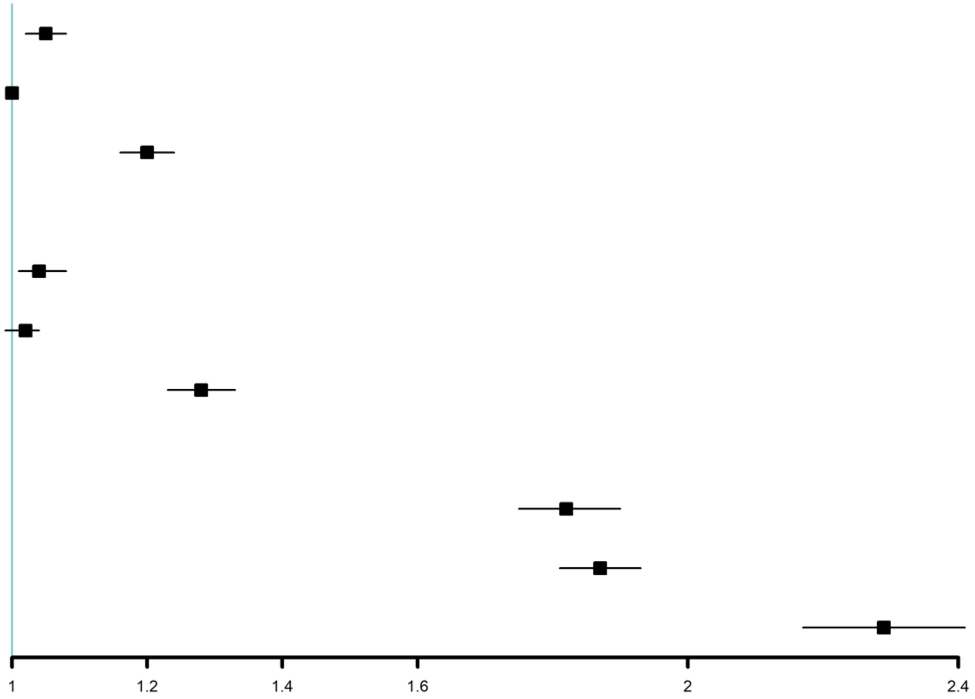


b) Joint effect of sleep disorder and glycemic status on all-cause mortality

| Group | HR (95%CI) | P value |
|---|---|---|
| **Normoglycemia** | | |
| No sleep disorder | reference | reference |
| Sleep disorder | 1.01 (0.98-1.04) | 0.4684 |
| **Prediabetes** | | |
| No sleep disorder | 1.01 (0.97-1.04) | 0.6509 |
| Sleep disorder | 1.04 (1.01-1.07) | 0.0153 |
| **Diabetes** | | |
| No sleep disorder | 1.78 (1.71-1.86) | <0.0001 |
| Sleep disorder | 1.92 (1.85-1.98) | <0.0001 |

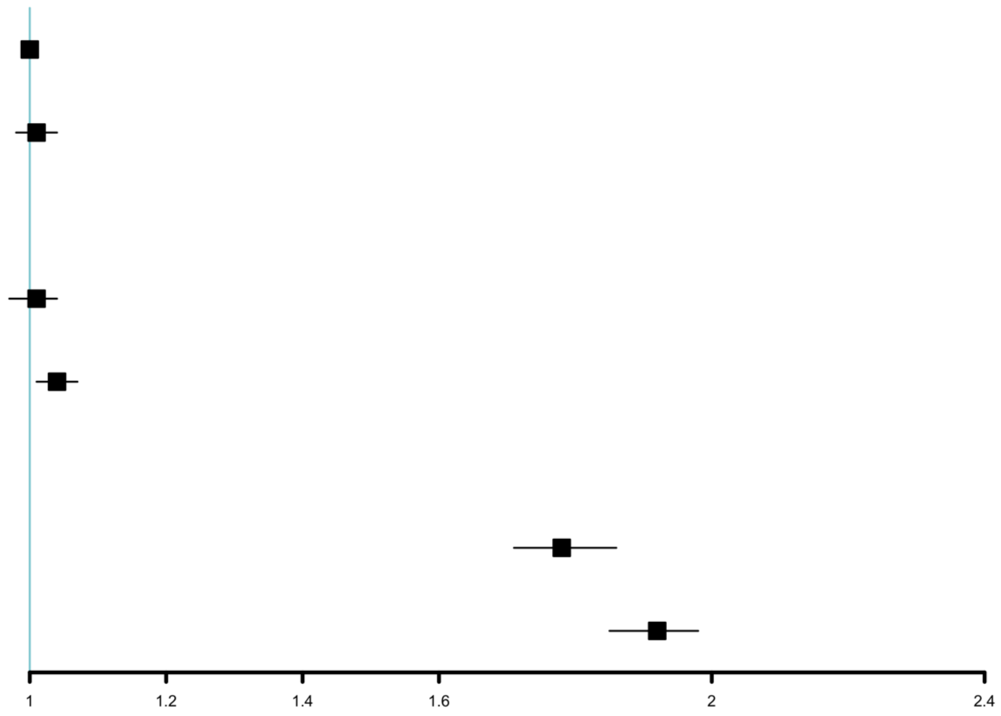

**Supplementary Figure 3** Joint association between sleep duration and glycemic status on all-cause mortality excluding participants who had cardiovascular disease or cancer at baseline

a) Joint effect of sleep duration and glycemic status on all-cause mortality

| Group | HR (95%CI) | P value |
|---|---|---|
| **Normoglycemia** | | |
| Less than 6 hours | 1.06 (1.03-1.09) | 0.0003 |
| 6-8 hours | reference | reference |
| More than 8 hours | 1.17 (1.12-1.21) | <0.0001 |
| **Prediabetes** | | |
| Less than 6 hours | 1.05 (1.01-1.09) | 0.0099 |
| 6-8 hours | 1.02 (0.99-1.05) | 0.2085 |
| More than 8 hours | 1.26 (1.20-1.32) | <0.0001 |
| **Diabetes** | | |
| Less than 6 hours | 1.86 (1.77-1.95) | <0.0001 |
| 6-8 hours | 1.91 (1.84-1.98) | <0.0001 |
| More than 8 hours | 2.27 (2.14-2.41) | <0.0001 |

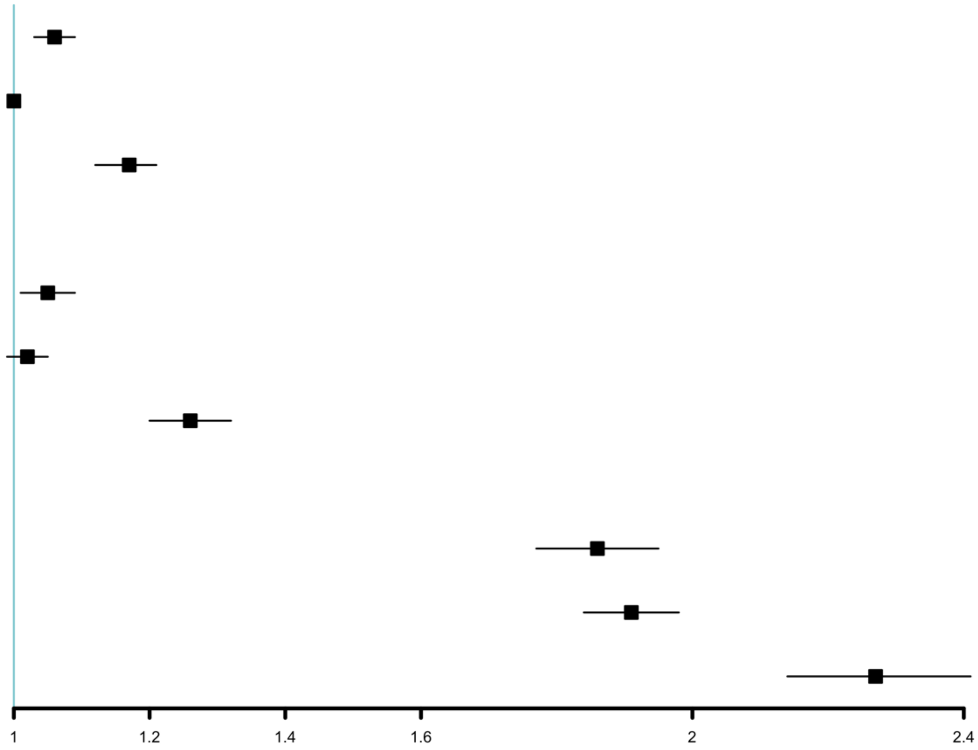

b) Joint effect of sleep disorder and glycemic status on all-cause mortality

| Group | HR (95%CI) | P value |
|---|---|---|
| **Normoglycemia** | | |
| No sleep disorder | reference | reference |
| Sleep disorder | 1.02 (0.99-1.04) | 0.4684 |
| **Prediabetes** | | |
| No sleep disorder | 1.00 (0.97-1.04) | 0.6509 |
| Sleep disorder | 1.04 (1.01-1.08) | 0.0153 |
| **Diabetes** | | |
| No sleep disorder | 1.78 (1.70-1.86) | <0.0001 |
| Sleep disorder | 1.96 (1.89-2.03) | <0.0001 |

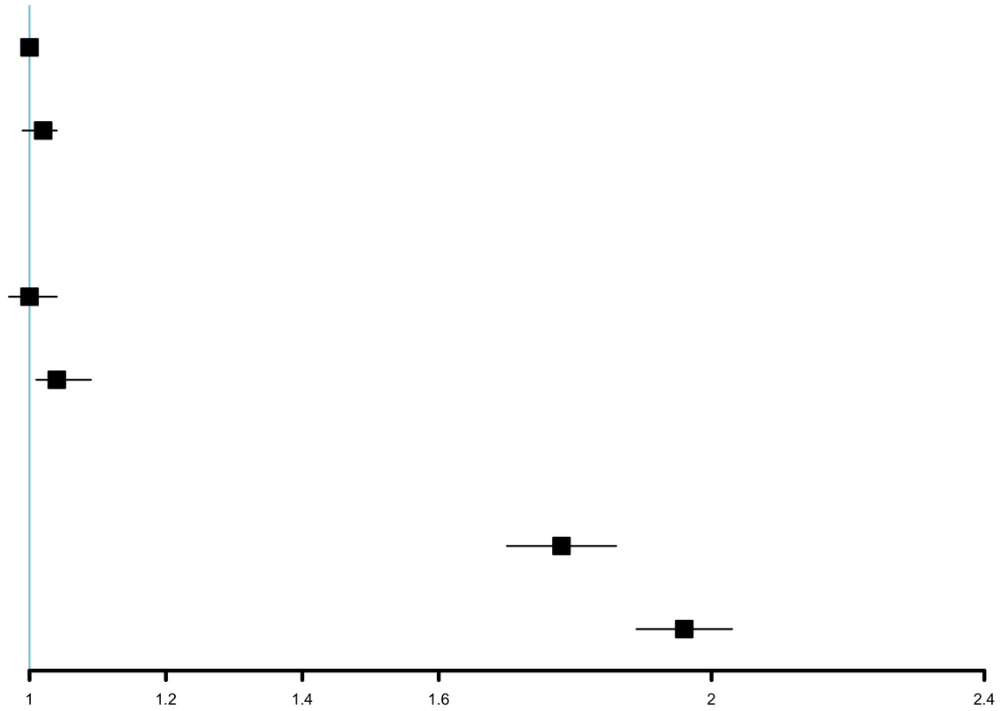

**Supplementary Figure 4** Joint association between sleep duration and glycemic status on all-cause mortality excluding participants with missing covariates

a) Joint effect of sleep duration and glycemic status on all-cause mortality

| Group | HR (95%CI) | P value |
|---|---|---|
| **Normoglycemia** | | |
| Less than 6 hours | 1.05 (1.01-1.08) | 0.0073 |
| 6-8 hours | reference | reference |
| More than 8 hours | 1.19 (1.15-1.24) | <0.0001 |
| **Prediabetes** | | |
| Less than 6 hours | 1.05 (1.01-1.09) | 0.0112 |
| 6-8 hours | 1.02 (0.99-1.05) | 0.1146 |
| More than 8 hours | 1.27 (1.21-1.33) | <0.0001 |
| **Diabetes** | | |
| Less than 6 hours | 1.81 (1.73-1.90) | <0.0001 |
| 6-8 hours | 1.89 (1.83-1.96) | <0.0001 |
| More than 8 hours | 2.24 (2.11-2.37) | <0.0001 |

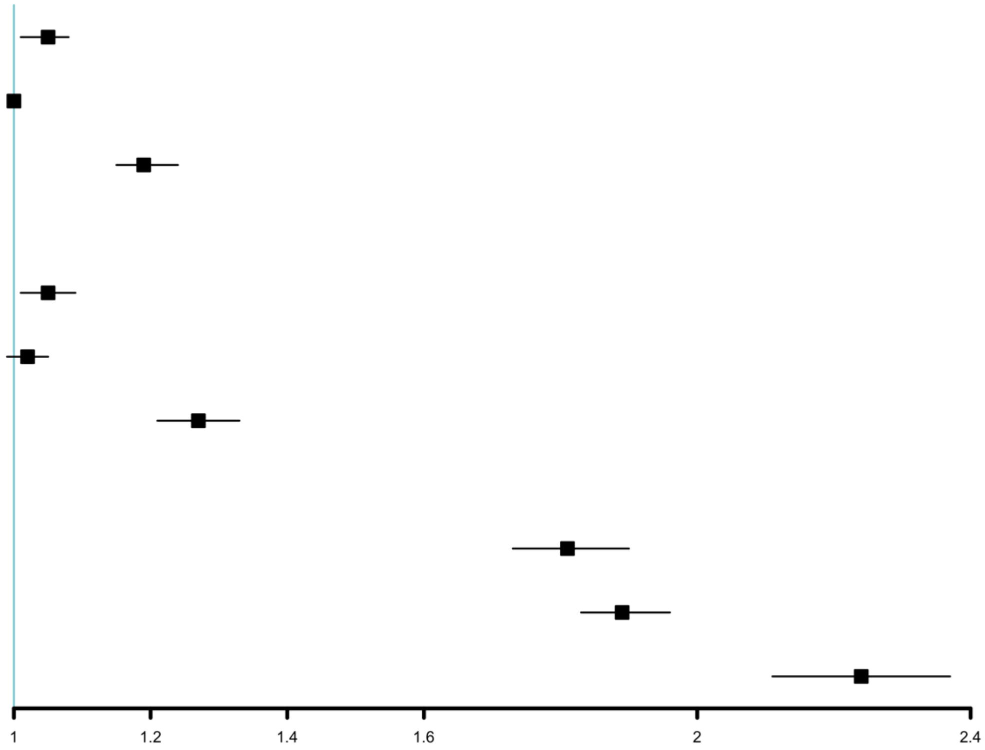

b) Joint effect of sleep disorder and glycemic status on all-cause mortality

| Group | HR (95%CI) | P value |
|---|---|---|
| **Normoglycemia** | | |
| No sleep disorder | reference | reference |
| Sleep disorder | 1.01 (0.98-1.04) | 0.5569 |
| **Prediabetes** | | |
| No sleep disorder | 1.01 (0.97-1.04) | 0.7104 |
| Sleep disorder | 1.04 (1.01-1.08) | 0.0081 |
| **Diabetes** | | |
| No sleep disorder | 1.76 (1.68-1.84) | <0.0001 |
| Sleep disorder | 1.92 (1.85-1.99) | <0.0001 |

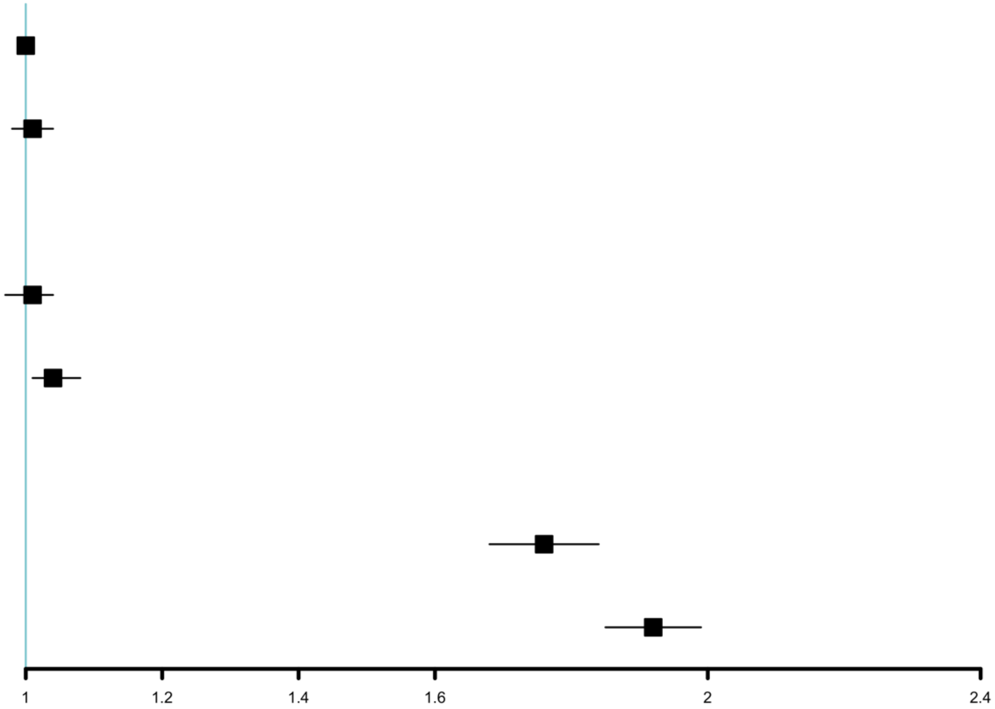

Supplement: Online Supplementary Document [file jogh-16-04002-s001.pdf]
